# Development and Implementation of a Perianesthetic Safety Checklist in a Veterinary University Small Animal Teaching Hospital

**DOI:** 10.3389/fvets.2018.00060

**Published:** 2018-04-03

**Authors:** Gwennaëlle Menoud, Shannon Axiak Flammer, Claudia Spadavecchia, Mathieu Raillard

**Affiliations:** Section of Anesthesiology and Pain Therapy, Department of Clinical Veterinary Medicine, Vetsuisse Faculty, University of Bern, Bern, Switzerland

**Keywords:** veterinary, anesthesia, checklist, Delphi method, implementation, perioperative, safety

## Abstract

**Introduction:**

The use of a surgical safety checklist is recommended by the World Health Organization and is associated with advantages: improved communication and reduced complications and mortality. Adapting checklists to the environment in which they are used improves their efficiency, but their implementation can be challenging. The aim of this study was to develop and implement a perianesthetic safety checklist for a small animal hospital.

**Materials and methods:**

A panel of eight anesthesia diplomates and seven residents and doctoral students were gathered. The Delphi method was used to generate a checklist. The checklist was presented individually to each user by the primary investigator and introduced into the clinical routine over a 5-week period. An interdisciplinary meeting was then held, and the checklist was modified further. Six months after introduction, the use of the checklist was directly observed during 69 anesthetic cases and a survey was sent to the users. A second implementation was organized after formally presenting the checklist to the staff, designating the anesthesia clinical lead as the person responsible for printing and controlling use of the checklist. A second evaluation was performed 3 months later (64 anesthetic cases).

**Results:**

Using the Delphi process led to the creation of a checklist consisting of three parts: “sign in” (before induction of anesthesia), “time out” (before the beginning of the procedure), “sign out” (at the end of the procedure). At the first assessment, the checklist was printed and used in 32% of cases and not printed in 41% of cases. Response rate of the survey was fair (19/32 surveys): 14/19 users thought the checklist contributed to improving communication; 15/19 reported improved safety and better management of the animals; 9/19 users avoided mistakes (77% would have omitted the administration of antimicrobial prophylaxis); 10/19 thought it was time consuming. At the second assessment, the checklist was used in 45% of cases (printed but not used in 55%). The use of the sign-out section of the checklist was significantly improved.

**Conclusion and clinical relevance:**

This study illustrates an innovative use of the Delphi method to create a safety checklist. Challenges associated with implementation are reported.

## Introduction

Safety checklists are designed to help prevent human errors in complex and high intensity working environments (1). The use of perioperative checklists was shown to reduce mortality and complication rates (2), improve communication and perception of safety in human hospital anesthesia teams (3), and reduce the incidence and severity of complications in veterinary settings (4).

Although a valid anesthesia checklist has been made available by the Association of Veterinary Anaesthetists (AVA),^1^ no checklist is universal because critical steps might differ from one institution to another. The AVA checklist does not address the specific safety issues of a large referral practice and therefore, the checklist should be adapted (5, 6).

The Delphi method was first developed by Dalkey and Helmer to obtain a reliable opinion consensus on specific topics (7) by gathering a group of experts to answer questions in three or more rounds. The method was designed to provide consensus in situations where there is conflicting scientific evidence or disagreements (8). Initially, the organizing team collects key questions on the topic of interest and selects suitable experts. In the first round, the experts are invited to express their opinion or to answer specific questions. These opinions or answers are grouped under a limited number of statements. In the second round, each expert ranks the statements in order of importance. Rankings are then summarized. In the third round, after considering the group’s response, the experts re-rank each statement and can change their initial ranking. The re-rankings are summarized, and the degree of consensus is assessed. If the degree of consensus is acceptable, the process ceases, if not, the third round is repeated until consensus is achieved. The Delphi method has been used by Tscholl et al. to generate a perianesthetic checklist in a human hospital (3). Applying the Delphi method to develop a perianesthetic checklist for a veterinary teaching hospital might represent an efficient way to obtain an accurate and robust instrument within a short time frame.

Once developed for a specific environment, a safety checklist has to be integrated into the daily clinical routine. This challenging step needs to be planned carefully, as it demands time and commitment from the entire team (5, 6).

The aims of the present study were: (i) to develop a veterinary perianesthetic safety checklist using the Delphi method; (ii) to plan and subsequently evaluate the implementation of this instrument in the clinical routine of a small animal teaching hospital.

## Materials and Methods

All veterinary anesthesiologists of the Vetsuisse Faculty (University of Bern and Zurich) were invited *via* email to participate in a specialist meeting. The meeting was scheduled for the day that allowed the highest number of participants. The veterinary perianesthetic safety checklist was designed using the World Health Organization (WHO) surgical safety checklist (9) as a model. Three main sections were envisaged: “sign in” (before induction of anesthesia), “time out” (before the beginning of the procedure), and “sign out” (at the end of the procedure). The goal of the meeting was for the experts to agree on a limited number of items to include in each section of the checklist using the Delphi method. The checklist agreed upon at the completion of the third round, was proposed for clinical use in the small animal teaching hospital of the University of Bern.

This first version of the checklist was introduced over a 5-week period and the main investigator (GM) was available to assist users individually. At the end of this period, an evaluation form was distributed to all checklist users (anesthesia clinicians, residents, technicians) and an interdisciplinary meeting was held that included the checklist users and the surgery team. Based on the feedback, a final version of the checklist was created. It was made available in the anesthesia induction area and users were informed orally about the availability of the new checklist. The checklist remained with the animal, kept by the anesthetist together with the anesthesia record throughout the procedure.

Six months later, a 17 question online survey^2^ (Data Sheet S1 in Supplementary Material) was sent per email to the anesthetists and the surgeons of the small animal hospital on clinics or having recently used the checklist (32 persons including veterinarians and technicians). It was created using an adaptation of the Safety Attitude Questionnaire (SAQ), an instrument developed to measure perceptions and attitudes in safety-related domains in health care (10), to assess the opinion of the checklist users.

Additionally, during a 3-week period, the main investigator (Gwennaëlle Menoud) observed the use of the checklist in clinical cases using a standard evaluation form (Data Sheet S2 in Supplementary Material). The observation started with the first surgical case of the day and continued according to the daily schedule in order to follow the highest possible number of cases; therefore, case selection was random. The main investigator verified the use of the checklist and noted when items on the checklist were discussed, but not recorded. In addition, she recorded the identity of the checklist user and surgical team, any reluctance to discuss the checklist, and the duration of the “time-out.”

**Figure 1:**
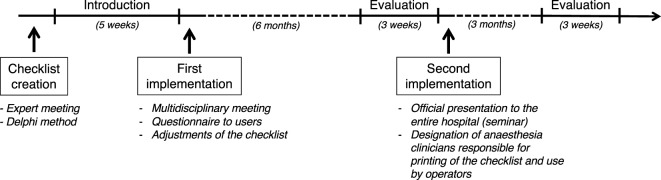
Time line of development, implementation, and evaluation of a perianesthetic safety checklist in the Small Animal Teaching hospital of the University of Bern. The checklist was created in an expert meeting using the Delphi method. The checklist was introduced in the clinics over a 5-week period. After introduction, a survey among the users was made and a multidisciplinary meeting was held, allowing the checklist to be adjusted to practice. Six months later, an evaluation of the use of the checklist was performed over a 3-week period. The tool was implemented a second time following oral introduction of the tool to the team and the designation of a responsible person. A second evaluation was performed 3 months later.

Based on the results of the online survey and direct observations, a second implementation phase was deemed necessary. It was decided that the lead anesthesia clinician (one person per day) would be responsible for printing the checklist and ensuring that all staff members would use it. All lead anesthesia clinicians were informed *via* email and during the monthly team meeting. Furthermore, the entire staff of the small animal teaching hospital (clinicians, residents, interns, students, technicians) were invited to a formal oral presentation illustrating the background, usefulness, and correct use of the checklist (including demonstration videos). The pitfalls and causes of failed implementation were discussed to raise user awareness. Three months later, a second evaluation was conducted over 3 weeks, by the main investigator, using the same methodology as previously described. Figure 1 illustrates the time line of development and implementation of this safety checklist.

Descriptive statistics were used to summarize the data and a Chi-square test was used to compare checklist use before and after the second implementation phase. SigmaPlot for Windows version 10.0 (Systat Software Inc., San Jose, CA, USA) was used for the analysis and statistical significance was set at *p* < 0.05.

## Results

The first expert meeting took place on 22/01/2016 at the University of Bern. A panel of eight diplomates of the European or American College of Veterinary Anesthesia and Analgesia (ECVAA/ACVAA) and seven residents and doctoral students from the veterinary anesthesia sections of the Universities of Bern and Zurich (Switzerland) were gathered.

A first version of the perianesthetic safety checklist was successfully generated using the Delphi method. The two items retained in the “sign in” part of the checklist were: (i) the verification of the animal’s identity and (ii) the responsible veterinarian. The panel agreed that it was the responsible veterinarian’s responsibility to (i) remain available throughout the procedure and (ii) ensure that the owner gave informed consent for general anesthesia before the procedure so these items did not need to be checked. Four points were highlighted by the Delphi method as equally important in the “time out” section: (i) the introduction of all persons present in the operating room, (ii) the confirmation of the animal’s identity, (iii) a clear discussion between the anesthetists and surgeons regarding possible complications; and (iv) the verification of administration of appropriate antimicrobial prophylaxis. In the “sign out” section, two items were retained: (i) the postoperative plan and (ii) the recovery organization. The palpation and emptying of the urinary bladder was considered an important complementary item and was, therefore, included in the checklist, because it was often forgotten and important for the animal’s comfort. Following the Delphi, it appeared that the most salient safety issues in our hospital were associated with the suboptimal communication between anesthesia and surgery teams at key time points. This is why, from this step on, the surgery team was present for every decision.

**Figure 2:**
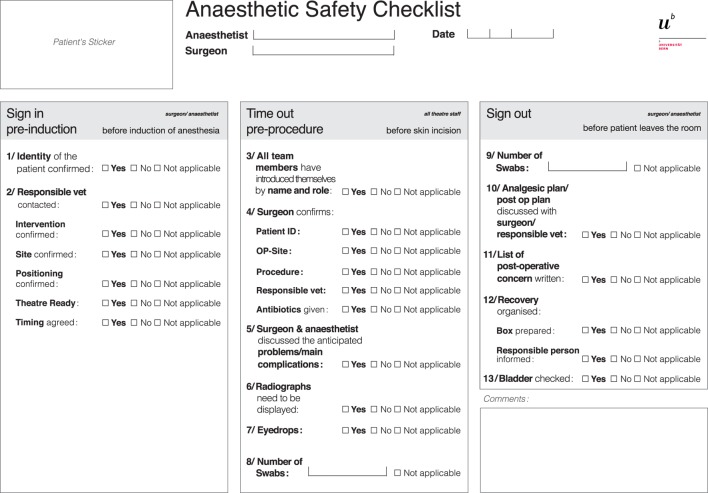
Perianesthetic safety checklist from the small animal teaching hospital of the University of Bern, created in expert meetings using the Delphi method and adapted after a 5-week introduction period and a multidisciplinary meeting.

Following the 5-week introduction phase, evaluations were collected. A multidisciplinary meeting including eight surgeons and nine anesthetists, who had used the checklist, contributed to its further adjustment. Elements added to the “time out” section were (i) the display of preoperative radiographs, (ii) the administration of eyedrops, and (iii) the number of swabs available. In the “sign out” section, the swab count was added. The final version of the perianesthetic safety checklist is presented in Figure 2.

The response rate to the online survey regarding the final checklist version was fair (19/32 respondents). On a scale from 1 (yes) to 5 (no), 14/19 users thought the checklist contributed to improved communication between surgeons and anesthetists (nine gave a score of 1, five a score of 2); 14/19 reported improved safety and management of the animals (seven respondents scored 1 and seven scored 2); nine users avoided mistakes because of the checklist (all would have omitted the administration of antimicrobial prophylaxis); and 10/19 respondents thought it was time consuming (six respondents scored 1 and four scored 2). Eight users answered the optional section question “Is the checklist used? If not, why?” (possibility to add multiple comments in the option “other”). Three users reported to have no time; one user reported that the checklist was not useful; and one user was unaware of its existence. Two users complained about the unavailability of printed checklists. One user did not use it for short and simple cases. Two users reported they forgot about it in emergency situations.

During the first 3-week evaluation period, direct observations were carried out on 69 anesthetic cases (37/69 were cases undergoing surgery). There were a total of 211 small animals that underwent general anesthesia during that period. The checklist was used in 22/69 (32%) cases, not printed in 28/69 (41%) cases, and printed but not used in 19/69 (27%) cases. The “sign in,” “time out,” and “sign out” sections were filled out in 14/69 (20%), 32/69 (46%), and 10/69 (14%) cases, respectively. Of the 32 cases in which the “time out” was discussed, 14/32 (44%) were discussed but not written down on the form, whereas 18/32 (56%) were both discussed and recorded. Information exchange during the “time out” was minimal (less than five items discussed) in 2/32 (6%) cases, moderate (between 5 and 8 items discussed) in 17/32 (53%) cases and satisfactory (>8 items discussed) in 13/32 (41%) cases. The average duration of the “time out” was 25 s.

During direct observation of the second implementation, 64 anesthetic cases were assessed (30/64 were followed by surgery). There were a total of 195 small animals that underwent general anesthesia during that period. The checklist was printed in all cases and used in 29/64 (45%) cases. Overall, the “sign in,” the “time out,” and the “sign out” were discussed in 17/64 (27%), 29/64 (45%), and 16/64 (25%) cases, respectively. When anesthesia was followed by surgery, the “sign in,” “time out,” and “sign out” were filled out in 17/30 (57%), 29/30 (97%), and 16/30 (53%), respectively. Of the 29 cases in which the “time out” was discussed, 1/29 (3%) was discussed but not written on the form, whereas 28/29 (97%) were also written down. Information exchange during the “time out” was minimal in 2/30 (7%) cases, moderate in 3/30 (10%) cases, and satisfactory in 25/30 (83%) cases. The average duration of the “time out” was 16 s.

The checklist was printed more after the second implementation (*p* = 0.001). There was no difference in its overall use after the second implementation (*p* = 0.158), but the “time out” was recorded more (*p* = 0.001). The use of the “sign out” section improved after the second implementation (*p* = 0.047).

## Discussion

The Delphi method allowed efficient selection of the items to include in the first version of the perianesthetic safety checklist. Indeed, only minor adjustments were necessary to finalize the checklist, once clinical experience had been gathered. Conversely, the introduction of the checklist into the clinical routine was difficult despite the planned implementation. Multiple interventions were required to optimize it. Communication did improve and this was verified by the observation that the “time out” was performed in almost all cases after the second implementation; information exchange was also efficient (more items discussed in a shorter time). Furthermore, based on user feedback, it is likely that the checklist contributed to more regular administration of antimicrobial prophylaxis but the general impact on perioperative safety could not be evaluated.

Checklists should be adapted to the setting in which they are used in Ref. (1, 11). We developed our checklist on the model of the WHO surgical safety checklist, which has proven its efficacy in increasing safety in human care (2), and kept its general tripartite structure. The Delphi method has already been proposed as a suitable method in development of a perianesthetic safety checklist in a human hospital (3), but a multidisciplinary meeting was necessary to adapt it further to our setting. The final version of the checklist is short, straightforward, and comprehensive. These properties are supposed to facilitate integration into the hospital’s routine (12) and reflect steps identified as critical to perianesthetic safety in the clinical routine.

The first implementation of the checklist was not successful in terms of compliance. Several reasons were identified: (i) the lack of a responsible person for the checklist; (ii) the frequent lack of printed copies of the checklist; and (iii) the use of the checklist for all anesthesia cases despite a design best suited for surgical procedures. These reasons probably contributed to the fact that users did not feel involved. A first important change, at the second implementation, consisted of designating responsible people for the printing and the distribution of the checklist. Defining rules and responsibilities were found to be essential in this context (13). Conley et al. mention that it is important to explain to the team members the aim and the use of the checklist before they start using it (13). If an implementation is imposed without introduction, it can be interpreted as constraint and restriction on the freedom of practice (14). If users are not aware of the checklist’s benefits and appropriate way of use, they might be uninterested or frustrated (13). In fact, half of the first survey respondents complained that the checklist was time consuming, when in fact time lost during the “time out” discussion was reasonable (25 s). It is likely that the initial introduction of the tool to the entire staff was not efficient enough to be taken seriously in our hospital. Our intention was to correct this with a formal oral presentation to the entire staff. In a normal working day in our small animal hospital, “on” staff includes approximately 20 veterinarians, 25 technicians, and 5–15 final year students (the entire staff being double this); all were invited to the presentation.

Different strategies have been proposed to improve staff member compliance including improved visibility of the checklist such as hanging posters in the operating rooms (14) or adding pink “time out” flyers to the sterile packs (14). Other advertising methods could also be considered such as announcements in the hospital newsletter and website, emails, or the display of the checklist as a screen saver. To date, we have not yet decided our next measures.

The timely administration of prophylactic antibiotics was shown to increase with the use of a safety checklist in some studies (15). The results of our survey show that many respondents had remembered to administer the antimicrobials thanks to the checklist. This could be considered an improvement in safety.

This study had several limitations. First, we had no quantification of the complication rates prior to the checklist introduction, which precludes conclusions on its real benefit in terms of safety. Second, it is possible that the users of the checklist recognized the primary investigator and that her presence influenced the use of the checklist during the periods of clinical evaluation. Third, in many instances, some items of the checklist were actually controlled but not recorded on the document meaning that some data could not be evaluated.

## Conclusion

The Delphi method can be used to generate a veterinary perianesthetic safety checklist. Responsible persons and clear communication of aim and expectations of the checklist are important when introducing a checklist in the clinical routine. Habits of a university veterinary teaching hospital can be changed, but implementation of a perianesthetic checklist can be a challenging process.

## Author Contributions

GM: data collection, analysis, interpretation, and redaction of the paper. SF and CS: study design, data analysis and interpretation, and redaction of the paper. MR: study design, data collection, analysis, interpretation, redaction of the paper.

## Conflict of Interest Statement

The authors declare that the research was conducted in the absence of any commercial or financial relationships that could be construed as a potential conflict of interest. The handling Editor and reviewer KI declared their involvement as co-editors in the Research Topic and confirmed the absence of any other collaboration.

## Footnotes

^1^https://ava.eu.com/wp-content/uploads/2015/11/AVA-Anaesthetic-Safety-Checklist-FINAL-UK-WEB-copy-2.pdf (Accessed: March 22, 2018).

^2^https://www.google.com/forms (Accessed: March 22, 2018).

## Supplementary Material

Data Sheet S1 Individual evaluation of the checklist by the user (anesthetist and surgeon).

Data Sheet S2 Evaluation form used to evaluate the three parts of the anesthesia procedure.
