# Supplementary material for: Development and Implementation of a Perianesthetic Safety Checklist in a Veterinary University Small Animal Teaching Hospital
Source: Front Vet Sci. 2018 Apr 3;5:60. doi: 10.3389/fvets.2018.00060 (PMC5891598; doi:10.3389/fvets.2018.00060)
Supplement: Data Sheet S1 — Individual evaluation of the checklist by the user (anesthetist and surgeon). [file data_sheet_1.PDF]

UNIVERSITÄT BERN

Evaluation of the Safety Checklist

*Mandatory

Name: * ______________________________

Use of the checklist* *Only one answer possible*

	1	2	3	4	5	
yes	○	○	○	○	○	no

If not, why? *Only one answer possible*

- no time
- not accurate
- too long
- not aware of its existence
- not available
- other(s):

Easy to use * *Only one answer possible*

	1	2	3	4	5	
yes	○	○	○	○	○	no

Easy to understand * *Only one answer possible*

	1	2	3	4	5	
yes	○	○	○	○	○	no

Time consuming * *Only one answer possible*

	1	2	3	4	5	
yes	○	○	○	○	○	no

Helps the communication with the surgeon/anesthetist* *Only one answer possible*

	1	2	3	4	5	
yes	○	○	○	○	○	no

Improvement of the management of the patient * *Only one answer possible*

	1	2	3	4	5	
yes	○	○	○	○	○	no

Improvement of the safety of the patient * *Only one answer possible*

	1	2	3	4	5	
yes	○	○	○	○	○	no

Have you avoided mistake(s), thanks to the checklist * *Only one answer possible*

	1	2	3	4	5	
yes	○	○	○	○	○	no

If yes, why? *Only one answer possible*

- wrong patient
- antibiotics forgotten
- wrong side
- other(s):

Is the "sign in" done in every case * *Only one answer possible.*

	1	2	3	4	5	
yes	○	○	○	○	○	no

If not, why? *Only one answer possible*

- no time (emergency)
- not accurate
- omission
- difficulty to communicate with surgeon/anesthetist
- other(s):

Is the “time out” done in every case * *Only one answer possible*

	1	2	3	4	5	
yes	○	○	○	○	○	no

If not, why? *Only one answer possible*

- no time (emergency)
- not accurate
- omission
- difficulty to communicate with surgeon/anesthetist
- other(s):

Is the “sign out” done in every case * *Only one answer possible*

	1	2	3	4	5	
yes	○	○	○	○	○	no

If not, why? *Only one answer possible*

- no time (emergency)
- not accurate
- omission
- difficulty to communicate with surgeon/anesthetist
- other(s):
