# Supplementary material for: Development and Implementation of a Perianesthetic Safety Checklist in a Veterinary University Small Animal Teaching Hospital
Source: Front Vet Sci. 2018 Apr 3;5:60. doi: 10.3389/fvets.2018.00060 (PMC5891598; doi:10.3389/fvets.2018.00060)
Supplement: Data Sheet S2 — Evaluation form used to evaluate the three parts of the anesthesia procedure. [file data_sheet_2.docx]

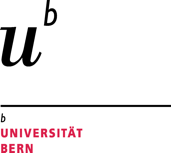


Assessment of the Perianesthetic Safety Checklist

**Date:____/ ____/________ Procedure/operating room: ______________________________________**

**Team members:**

**Surgeon: __________________________**

**Anesthetist:_________________________**

**Nurses: ____________________________**

# Sign-in

When was the sign-in conducted?

- - Before induction
  - After induction
  - Other

|  | | Not checked | Check verbal | Check written |
| --- | --- | --- | --- | --- |
| Identity of the patient | |  |  |  |
| Responsible vet | Intervention |  |  |  |
|  | Site |  |  |  |
|  | Positioning |  |  |  |
|  | Theater ready |  |  |  |
|  | Timing agreed |  |  |  |
|  |  |  |  |  |

Items checked verbally/written

Who participated in the sign-in? ____________________________________________

- 1. Who initiated the sign-in? ____________________________________________
  2. Who led the sign-in? ________________________________________________

# Time-out

How long did the time-out take? _____________________________________________

When was the time-out conducted?

- - Before skin incision
  - After skin incision
  - Other

Who participated in the time-out? ___________________________________________

Were all team members (Surgeon, anesthetist, nurse) present for the time-out?

- - - yes
    - no (who was missing? ________________________________________)

Who initiated the time-out? _________________________________________________

Who led the time-out? ______________________________________________________

How well was the information exchanged?

- - - Minimal exchange of information
    - Moderate exchange in information
    - All relevant information exchanged
    - No exchange of information

Was there any resistance?

- ***Yes***
- From surgical team
- From anesthesia team
- From nurse (TPA)
- ***No***

Items checked verbally/written

|  | | Not checked | Check verbal | Check written |
| --- | --- | --- | --- | --- |
| All team members have introduced themselves by name and role | |  |  |  |
| Surgeon confirms | Patient ID |  |  |  |
|  | Op-site |  |  |  |
|  | Procedure |  |  |  |
|  | Responsible vet |  |  |  |
|  | Antibiotics given |  |  |  |
| Anticipated problem/main complications discussed | |  |  |  |
| Radiographs need to be displayed | |  |  |  |
| Number of swab | |  |  |  |

# Sign-out

When was sign-out completed?

- - Before closing the skin
  - During closing of the skin
  - After closing the skin

Items checked verbally/written

|  | | Not checked | Check verbal | Check written |
| --- | --- | --- | --- | --- |
| Number of swabs | |  |  |  |
| Analgesic plan discussed with surgeon/responsible vet | |  |  |  |
| List of postoperative concerns written | |  |  |  |
| Recovery organized | Box prepared |  |  |  |
|  | Responsible person informed |  |  |  |
| Bladder checked | |  |  |  |

Who participated in the sign-out? ___________________________________________

Were all team members (Surgeon, anesthetist, nurse) present?

- - yes
  - no (if no, who was missing? __________________)

Who initiated the sign-out? _________________________________________________

Who led the sign-out? _____________________________________________________

Was there any resistance to the sign-out?

- No
- Yes (from: ________________________________)

Comment? _______________________________________________________________
